# Effects of liraglutide on astrocyte polarization and neuroinflammation in db/db mice: focus on iron overload and oxidative stress

**DOI:** 10.3389/fncel.2023.1136070

**Published:** 2023-06-01

**Authors:** Ji-Ren An, Jun-Tong Liu, Xiao-Meng Gao, Qing-Feng Wang, Gui-Yan Sun, Jia-Nan Su, Chi Zhang, Jia-Xiang Yu, Yu-Feng Yang, Yan Shi

**Affiliations:** ^1^Liaoning Key Laboratory of Chinese Medicine Combining Disease and Syndrome of Diabetes, Liaoning University of Traditional Chinese Medicine, Shenyang, China; ^2^College of Integrative Chinese and Western Medicine, Hebei University of Chinese Medicine, Shijiazhuang, China

**Keywords:** diabetic cognitive impairment, hippocampus, neuroinflammation, astrocyte polarization, iron overload, oxidative stress

## Abstract

Neuroinflammation plays a crucial role in the occurrence and development of cognitive impairment in type 2 diabetes mellitus (T2DM), but the specific injury mechanism is not fully understood. Astrocyte polarization has attracted new attention and has been shown to be directly and indirectly involved in neuroinflammation. Liraglutide has been shown to have beneficial effects on neurons and astrocytes. However, the specific protection mechanism still needs to be clarified. In this study, we assessed the levels of neuroinflammation and A1/A2-responsive astrocytes in the hippocampus of db/db mice and examined their relationships with iron overload and oxidative stress. First, in db/db mice, liraglutide alleviated the disturbance of glucose and lipid metabolism, increased the postsynaptic density, regulated the expression of NeuN and BDNF, and partially restored impaired cognitive function. Second, liraglutide upregulated the expression of S100A10 and downregulated the expression of GFAP and C3, and decreased the secretion of IL-1β, IL-18, and TNF-α, which may confirm that it regulates the proliferation of reactive astrocytes and A1/A2 phenotypes polarize and attenuate neuroinflammation. In addition, liraglutide reduced iron deposition in the hippocampus by reducing the expression of TfR1 and DMT1 and increasing the expression of FPN1; at the same time, liraglutide by up-regulating the levels of SOD, GSH, and SOD2 expression, as well as downregulation of MDA levels and NOX2 and NOX4 expression to reduce oxidative stress and lipid peroxidation. The above may attenuate A1 astrocyte activation. This study preliminarily explored the effect of liraglutide on the activation of different astrocyte phenotypes and neuroinflammation in the hippocampus of a T2DM model and further revealed its intervention effect on cognitive impairment in diabetes. Focusing on the pathological consequences of astrocytes may have important implications for the treatment of diabetic cognitive impairment.

## 1. Introduction

There is ample evidence that diabetes mellitus (DM) is strongly associated with a decline in cognitive function and damage to brain structure. Cognitive deficits are revealed in the earliest stages of diabetes and worsen with continued poor glycemic control (Zilliox et al., 2016). People with long-term type 2 diabetes mellitus (T2DM) had a 37% or more increased risk of dementia, an extremely severe form of neurodegeneration (Biessels et al., 2006; Smolina et al., 2015). Considering the serious impairment of the quality of daily life imposed by cognitive deficits, effective intervention is necessary.

Notably, DM-induced central nervous system (CNS) inflammation is a risk factor for the development and progression of cognitive impairment (Muriach et al., 2014), in which hippocampal neuroinflammation plays a key role (Dinel et al., 2011; Feng et al., 2017). High glucose promotes the secretion of proinflammatory cytokines (IL-6, IL-1β, and TNF-α) and decreases the expression of brain-derived neurotrophic factor (BDNF) in hippocampal neurons cultured *in vitro*, which may be an important factor leading to neuronal and synaptic plasticity damage (Dinel et al., 2011; Xu et al., 2021). In the hippocampus of diabetic mice, the inflammatory response was accompanied by activation of astrocytes, which may influence or exacerbate the neuronal damage process (Nagayach et al., 2014). Therefore, it is necessary to further study the role of astrocytes in DM-induced neuroinflammation.

Normal maintenance of neurological function requires the support of astrocytes, which provide structural and metabolic support to neurons and regulate central nervous homeostasis (Colombo and Farina, 2016; Durkee and Araque, 2019). Interestingly, astrocyte polarization has recently been shown to be involved in the pathophysiology of hippocampal neuroinflammation. CNS injury can stimulate astrocyte proliferation, activation, conversion into reactive astrocytes, and polarization into neurotoxic A1-reactive astrocytes and neuroprotective A2-reactive astrocytes (Miyamoto et al., 2020). Type A1 cells lose the basic function of astrocytes and release or produce factors that contribute to neurotoxicity and inflammation, resulting in the death of neurons and oligodendrocytes; type A2 cells can secrete a variety of neurotrophic factors and play a neuroprotective role in CNS diseases (Liddelow et al., 2017). This drew our attention to the altered polarization of hippocampal astrocytes, which may further our understanding of astrocytes in diabetic conditions.

Often, hyperglycemic neurodegeneration and chronic inflammatory responses are induced by excess reactive oxygen species (ROS) and reactive nitrogen species (RNS) (Mello et al., 2012; Oliveira et al., 2016). The imbalance of the oxidation/antioxidant system is related to the activation of astrocytes and the proliferation of reactive astrocytes (Misra et al., 2020; Dragic et al., 2021; Darvish Khadem et al., 2022). It is well known that iron metabolism underlies the dynamic interaction between oxidants and antioxidants. However, excess iron can induce the generation of massive ROS by participating in the electron transport of the oxidative respiratory chain in mitochondria, leading to oxidative stress and lipid peroxidation and ultimately disrupting the normal functional state of cells (Imam et al., 2017). Astrocytes regulate brain iron uptake and are responsible for iron redistribution to neuronal cells. Specifically, iron enters neurons through transferrin receptor (TfR) and divalent cation transporter 1 (DMT1), while DMT1 is strongly expressed in the terminal foot of astrocytes and may be the main driver of iron uptake in astrocytes (Salvador, 2010; Vela, 2018; Cheli et al., 2020). Ferroportin 1 (FPN1) widely exists in neurons and various glial cells, and the release of iron in cells mainly depends on FPN1 (Batista-Nascimento et al., 2012). Recent evidence suggests that an inflammatory environment can promote astrocyte activation by increasing iron accumulation (Pelizzoni et al., 2013). Our previous study demonstrated iron accumulation in the hippocampus of T2DM models, which exacerbates oxidative damage (An et al., 2022).

Liraglutide (LIRA), a GLP-1 receptor agonist, is approved for the clinical treatment of obesity and T2DM (Tella and Rendell, 2015). Our research group and others have found that LIRA improves cognitive impairment, with both neuronal and astrocyte effects (Batista et al., 2018; Zheng et al., 2021; An et al., 2022). We hypothesized that LIRA ameliorates hippocampal A1/A2 astrocyte imbalance and weakens neuroinflammation in db/db mice, which might attenuate the exacerbation of diabetic cognitive impairment. To test this hypothesis, we evaluated behavioral performance and the expression of NeuN, BDNF and inflammatory cytokines in the hippocampus after LIRA treatment. At the same time, we observed that LIRA reversed the increased expression of complement component 3 (C3) and the decreased expression of S100A10, the most commonly used specific markers of A1/A2 astrocytes, in the hippocampus of db/db mice (Liddelow et al., 2017). We also assessed the status of iron overload and the expression of the associated proteins TfR1, FPN1, and DMT1 in the hippocampus of these mice while paying attention to the levels of hippocampal oxidative stress and lipid peroxidation, which affect astrocyte polarization.

## 2. Materials and methods

### 2.1. Animals

Eight-week-old male C57BL/6J mice (*n* = 10) and C57BL/KsJ db/db (db/db) mice (*n* = 20) were purchased from Changzhou Cavens Experimental Animal Co., Ltd. All mice were adaptively fed for 1 week. Animal care strictly followed the National Institutes of Health Guidelines for the Care and Use of Laboratory Animals. All animal experiments were approved by the Medical Ethics Animal Care and Use Committee of Liaoning University of Traditional Chinese Medicine (No. 2100004202129).

The db/db mice were randomly divided into the db/db group (db/db) and liraglutide group (LIRA). C57BL/6J mice were used as the control group (Con). In the LIRA group, liraglutide (200 μg/kg/day, HY-P0014, MedChemExpress) was injected intraperitoneally from the first day of the 10th week to the last day of the 14th week. An equal volume of 0.9% saline was injected for the Con group and db/db group.

### 2.2. Glycolipid metabolism detection

The mice were fasted for 12 h before sacrifice, and the fasting blood glucose (FBG) level was measured using blood collected from the tail vein.

Serum insulin (INS) and glycated hemoglobin A1c (HbA1c) were determined by an ELISA kit from Jiangsu Meimian Industrial Co., Ltd. (Yancheng, China); the measurement of serum total cholesterol (TC), triglyceride (TG), and low-density lipoprotein cholesterol (LDL-C) content was performed separately according to the kit instructions (Nanjing Jiancheng Bioengineering Institute).

Furthermore, glycogen in the hippocampus was detected using a glycogen content detection kit (KTB1340, Abbkine).

### 2.3. Morris water maze

Morris water maze (MWM) assessments, widely used to test memory function, were performed using the SMART-CS (PanLab, Barcelona, Spain) program as previously described (An et al., 2020). The platform was placed above the water (platform visible) for a day of memory training. From day 2 to day 6, the platform was placed below the water surface (hidden platform) for site navigation tests. Each mouse was placed into the tank from one of four different orientations, and the time delay to reach the platform was recorded. The average time spent in the four quadrants was calculated and counted as the escape latency. On day 7, the platform was removed for probe testing. The number of times the platform was crossed within 1 min was recorded.

### 2.4. Transmission electron microscopy analysis

The synaptic state of hippocampal neurons was visualized using transmission electron microscopy (TEM). We rapidly excised fresh hippocampal tissue pieces no larger than 1 mm^3^. The tissue was transferred to electron microscope fixative and fixed at 4°C. Afterward, the resin block was postfixed with 1% osmic acid, dehydrated at room temperature, infiltrated with acetone and 812 embedding agent, polymerized, and removed for later use. Next, the resin block was cut into 60–80 nm ultrathin sections by an ultramicrotome and stained. All operations were performed according to previous reports (An et al., 2022). Images were observed by a Hitachi HT7800 electron microscope.

### 2.5. IL-6, IL-1β, TNF-α, and IL-18 analysis

We used ELISA kits (Jiangsu Meimian Industrial Co., Ltd., Yancheng, China) to measure mouse serum interleukin 1 beta (IL-1β), interleukin 6 (IL-6), tumor necrosis factor-α (TNF-α), and interleukin 18 (IL-18) levels according to the manufacturer’s instructions.

In addition, the levels of IL-1β and IL-6 in the hippocampus were measured according to the manufacturer’s instructions using an ELISA kit (88-7013/88-7064, Invitrogen).

### 2.6. Hippocampal iron content detection

We also measured hippocampal iron levels using a tissue iron assay kit (A039-2, Nanjing Jiancheng Bioengineering Institute). A total of 10% hippocampal tissue homogenates were made, and the protein concentration was determined by the BCA method. Furthermore, the iron content of the tissue was measured according to the manufacturer’s instructions. The absorbance level at a wavelength of 520 nm was detected and calculated.

### 2.7. Hippocampal MDA, SOD, and GSH analysis

The contents of malondialdehyde (MDA), superoxide dismutase (SOD), and glutathione (GSH) were determined according to the kit instructions of Nanjing Jiancheng Bioengineering Institute. The content values were calculated according to the corresponding formula (A003-1/A001-1/A006-2, Nanjing Jiancheng Bioengineering Institute).

### 2.8. Perls’ staining

Perls’ staining was used to detect the content and distribution of iron in brain tissue, especially in the hippocampus. After dewaxing and rehydration, the sections were incubated with a mixture of H_2_O_2_ and methanol at room temperature for 30 min. Then, the slices were immersed in a mixture of fresh potassium ferricyanide and hydrochloric acid and incubated at room temperature without light for 12 h. Subsequently, DAB staining was performed, and the sections were sealed with neutral resin. The positive area was calculated by ImageJ software.

### 2.9. Dihydroethidium staining

Dihydroethidium (DHE) staining was used to detect ROS levels in the hippocampus, as described earlier (An et al., 2020). After washing the slices with 0.01 M phosphate buffered saline (PBS), the slices were incubated for 30 min with 5 μM DHE staining solution (19709, Cayman Chemical) in the dark at 37°C. Then, the slices were rinsed with PBS, sealed with anti-quenching fluorescence sealant, and observed under a fluorescence microscope. The positive area was calculated by ImageJ software.

### 2.10. Immunohistochemistry staining

The hippocampus was paraffin-embedded and sectioned, followed by immunohistochemical (IHC) staining. The sections were deparaffinized, rehydrated, and incubated with endogenous peroxidase blocking buffer for 20 min at room temperature. Then, citrate buffer (pH = 6.0) was added to the tissue sections for antigen retrieval, and then incubated with goat serum for 30 min at 37°C. Next, they were incubated with primary antibody against glial fibrillary acidic protein (GFAP, GB12096, Servicebio, Wuhan, China) at 4°C overnight. HRP-conjugated secondary antibody was added dropwise, and the tissue sections were incubated at 37°C for 1 h, enhanced with DAB, redyed with hematoxylin for 8 min, differentiated with hydrochloric acid ethanol for 5 s, dehydrated with ethanol, and mounted with neutral resin. The positive cell area was observed under a microscope. ImageJ software was used to analyze the positive area of GFAP staining in the hippocampus by determining the threshold area percentage. All sections used the same threshold parameter.

### 2.11. Immunofluorescence staining

Sections were deparaffinized and rehydrated, moreover citric acid buffer and goat serum were added for antigen retrieval and blocking, followed by primary antibodies against C3 (21337-1-AP, Proteintech, Wuhan, China), S100A10 (11250-1-AP, Proteintech, Wuhan, China), TNF-α (60291-1-Ig, Proteintech, Wuhan, China), DMT1 (60291-1-Ig, Proteintech, Wuhan, China), 4-hydroxynonenal (4-HNE, ARG23717, Arigo, Taiwan, China), and GFAP (GB12096, Servicebio, Wuhan, China) overnight at 4°C. A fluorescent secondary antibody, DAPI reaction solution, anti-fluorescence decay mounting medium, and mounting slide were added successively. The images were observed and collected by fluorescence microscope.

### 2.12. Western blot analysis

After the hippocampal tissues were thawed, RIPA lysis buffer was added, and they were homogenized and fully lysed. Then, the total protein in the supernatant was obtained by centrifugation; in addition, the total protein concentration was measured by a BCA kit (CW0014S, CoWin Biosciences, Taizhou, China). An equal amount of protein sample was added for SDS–PAGE and then transferred to nitrocellulose membranes. The membranes were blocked with 5% non-fat dry milk for 2 h. The blots were incubated with primary antibodies against NeuN (GB11138, Servicebio, Wuhan, China), BDNF (GB11559, Servicebio, Wuhan, China), GFAP (GB12096, Servicebio, Wuhan, China), C3 (21337-1-AP, Proteintech, Wuhan, China), S100A10 (11250-1-AP, Proteintech, Wuhan, China), IL-1β (AF5103, Affinity, Cincinnati, OH, USA), IL-18 (10663-1-AP, Proteintech, Wuhan, China), TNF-α (60291-1-Ig, Proteintech, Wuhan, China), L-ferritin (FTL, ab109373, Abcam, Cambridge, MA, USA), TfR1 (136890, Invitrogen, Carlsbad, CA, USA), DMT1 (60291-1-Ig, Proteintech, Wuhan, China), FPN1 (MTP11-A, Alpha Diagnostic International, San Antonio, TX, USA), NADPH oxidase 2 (NOX2, 19013-1-AP, Proteintech, Wuhan, China), NADPH oxidase 4 (NOX4, 14347-1-AP, Proteintech, Wuhan, China), superoxide dismutase 2 (SOD2, ARG54937, Arigo, Taiwan, China), β-actin (AF7018, Affinity, Cincinnati, OH, USA; GB15001, Servicebio, Wuhan, China), and GAPDH (GB15002, Servicebio, Wuhan, China) for 12 h at 4°C. The blots were incubated with HRP-conjugated secondary antibodies for 1.5 h at room temperature. Finally, the immunoreactive proteins were detected by enhanced chemiluminescence (ECL).

The resulting bands were quantified by VisionCapt v16.12. We selected each target protein of the membrane by setting the standard line and analyzed the data in the system. All analyses are based on the results of volume (which is obtained by multiplying the value of height and the value of area) as the standard, and the ratio of the value of the target protein to the value of the internal reference protein was determined. Finally, we used the ratio of the first Con sample on each strip as the standard value and compared the remaining ratios with it.

### 2.13. Statistical analysis

Statistical data were analyzed using GraphPad Prism 6.0 software and are expressed as the mean ± SEM. One-way ANOVA was used for statistical analysis, and then the LSD posttest was performed. *p* < 0.05 was considered significant.

## 3. Results

### 3.1. LIRA improves glycolipid metabolism disorder in db/db mice

**FIGURE 1:**
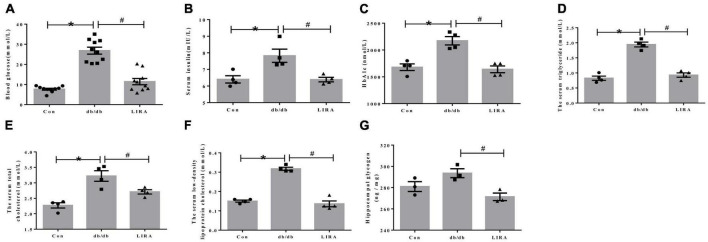
Liraglutide improves abnormal glucose and lipid metabolism in db/db mice. **(A)** Fasting blood glucose of mice (*n* = 10). **(B)** Serum insulin levels in each group (*n* = 4). **(C)** Glycated hemoglobin A1c of mice (*n* = 4). Serum triglyceride **(D)**, total cholesterol **(E)**, and low-density lipoprotein cholesterol **(F)** levels in mice (*n* = 4). **(G)** Glycogen content in the hippocampus (*n* = 3). The data are shown as the means ± SEMs. *p < 0.05 vs. Con group, ^#^*p* < 0.05 vs. the db/db group.

At the end of week 14, we measured blood glucose in all groups. FBG levels were significantly elevated in db/db mice and were effectively decreased in the LIRA group (Figure 1A). The data showed that LIRA effectively reversed the increase in INS and the increase in HbA1c levels in db/db mice (Figures 1B, C). At the same time, we assessed the levels of serum TC, TG, and LDL-C, which reflect changes in lipid metabolism. Compared with the Con mice, the serum TC, TG, and LDL-C levels were significantly increased in the db/db mice, and compared with the db/db mice, the above indicators were significantly decreased in the LIRA group (Figures 1D–F). Based on the above results, we once again confirmed the effect of LIRA in resolving the disturbance of glucose and lipid metabolism in db/db mice, consistent with previous findings.

Disturbance of glucose metabolism in different organs is a common symptom of diabetic complications. Furthermore, we detected the level of glycogen in the hippocampus. Compared with Con mice, db/db mice did not show significantly elevated glycogen levels in the hippocampus (Figure 1G). Interestingly, LIRA decreased glycogen content in the hippocampus of db/db mice (Figure 1G).

### 3.2. LIRA attenuates cognitive deficits and neuronal damage in db/db mice

**FIGURE 2:**
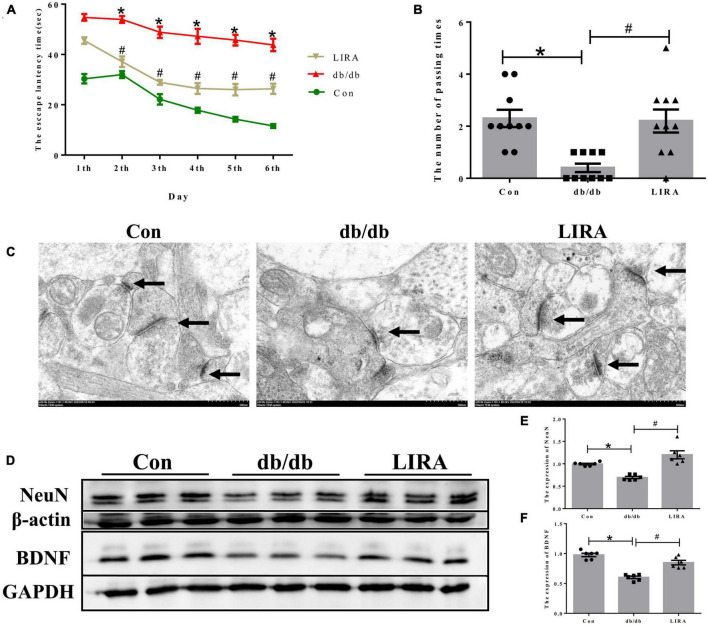
Liraglutide attenuates cognitive impairment and neuronal damage in db/db mice. **(A)** An escape latency assay was performed over 6 days (*n* = 10). **(B)** Number of platform crossings on day 7 (*n* = 10). **(C)** Neuronal synapses observed by TEM, as indicated by the arrows (scale bar: 1 μm, *n* = 3). **(D–F)** The expression and statistics of NeuN and BDNF proteins in the hippocampus (*n* = 6). The data are shown as the means ± SEMs. *p < 0.05 vs. Con group, ^#^*p* < 0.05 vs. the db/db group.

We tested the spatial learning and memory ability of mice by the MWM test. The escape latency was increased in the db/db group compared to that in the con group (Figure 2A). On the last day, the number of times the mice in the db/db group crossed the platform within 1 min was significantly reduced (Figure 2B). However, the prolonged escape latency was improved in the LIRA group, and the number of platform crossings was increased in the LIRA group (Figures 2A, B). Synaptic integrity was assessed by TEM. Compared to the postsynaptic density in the normal synaptic cleft of Con mice, the postsynaptic density was reduced in db/db mice (Figure 2C), while LIRA increased the postsynaptic density (Figure 2C).

Furthermore, we assessed the expression of NeuN and BDNF by Western blotting. NeuN, a reliable marker of mature neurons, was used to directly assess neuronal loss (Duan et al., 2016). BDNF, the most widely studied neurotrophic factor, has been identified as an essential regulator of normal brain development and neuronal plasticity that counteracts neuronal damage (Chen et al., 2017). The results showed that the expression levels of NeuN and BDNF in the hippocampus were lower in the db/db group than in the Con group, and they were partially restored after LIRA intervention (Figures 2D–F). Taken together, these results suggest that LIRA can effectively protect damaged neurons and cognitive impairment in db/db mice.

### 3.3. LIRA attenuates astrocyte activation

**FIGURE 3:**
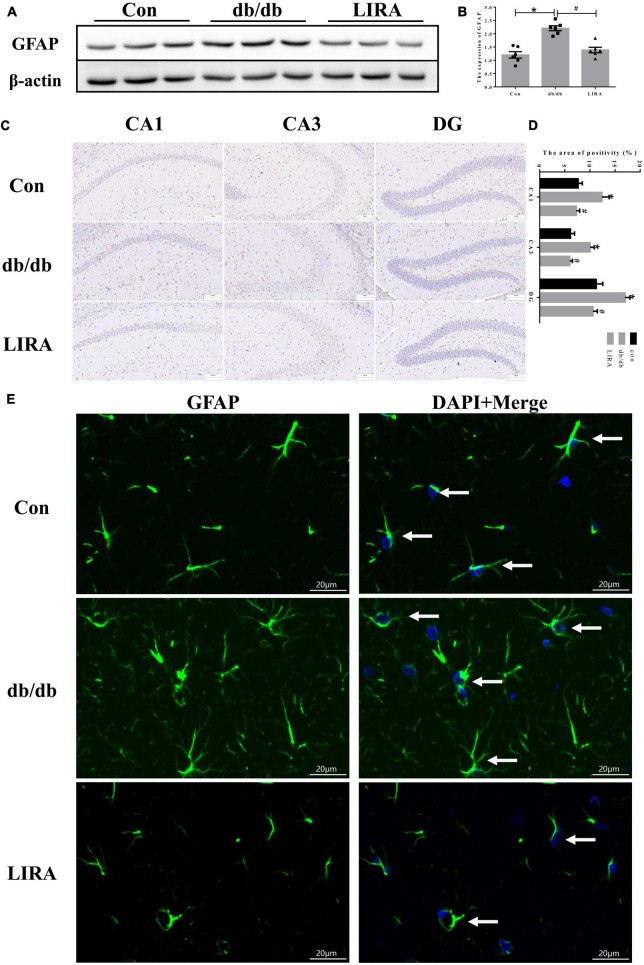
Liraglutide attenuates astrocyte activation. **(A,B)** The expression and statistics of GFAP proteins in the hippocampus (*n* = 6). **(C)** Immunohistochemical staining of GFAP proteins (scale bar: 100 μm, *n* = 3). **(D)** The GFAP-positive area, as shown in panel **(C)**. **(E)** Immunofluorescence staining of GFAP in the hippocampus, as indicated by the arrows (scale bar: 20 μm, *n* = 3). The data are shown as the means ± SEMs. *p < 0.05 vs. Con group, ^#^*p* < 0.05 vs. the db/db group.

Glial fibrillary acidic protein is a protein expressed in astrocytes and is an important part of the cytoskeleton. Some studies have confirmed that an increase in GFAP indicates the activation of astrocytes (Oliveira et al., 2016). During this period, astrocytes change their appearance and exhibit characteristic hypertrophy (Moghaddam et al., 2014). The results of Western blot analysis showed that db/db mice exhibited significantly higher GFAP expression in the hippocampus, and increased GFAP immunostaining was detected in the CA1, CA3, and dentate gyrus (DG) regions (Figures 3A–D). In addition, more astrocytes appeared to display slight hypertrophy and more cytoplasmic extensions (Figure 3E). LIRA treatment resulted in decreased GFAP expression and improved astrocyte morphology in db/db mice, which may represent a reduction in reactive astrogliosis (Figures 3A–E).

### 3.4. LIRA regulates the polarization of astrocytes

**FIGURE 4:**
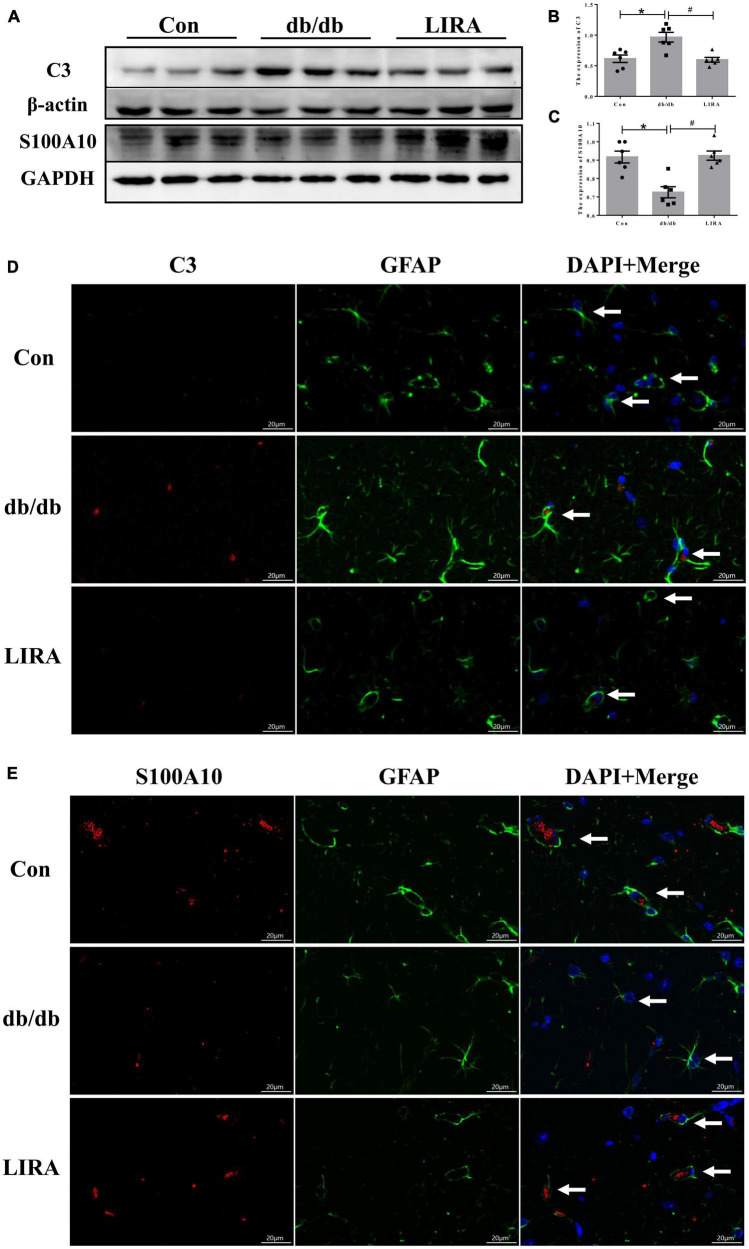
Liraglutide regulates the polarization of astrocytes. **(A–C)** The expression and statistics of C3 and S100A10 proteins in the hippocampus (*n* = 6). **(D,E)** Immunofluorescence double-labeled staining of C3/GFAP and S100A10/GFAP in the hippocampus, as indicated by the arrows (scale bar: 20 μm, *n* = 3). The data are shown as the means ± SEMs. *p < 0.05 vs. Con group, ^#^*p* < 0.05 vs. the db/db group.

As mentioned above, reactive astrocyte phenotypes can be converted to the “A1” and “A2” phenotypes (Liddelow et al., 2017). We examined the expression of C3 and S100A10, markers of A1/A2 reactive astrocytes, by Western blotting. Quantitative analysis showed that there were significantly more C3-expressing astrocytes in the hippocampus of db/db mice than in the Con group; in contrast, the expression of S100A10 was decreased in the hippocampus of db/db mice (Figures 4A–C). Noticeably, C3 overexpression was reduced and S100A10 expression was upregulated in the hippocampus of db/db mice treated with LIRA (Figures 4A–C).

Furthermore, we observed the morphological changes in astrocytes colabeled with C3 and GFAP or S100A10 and GFAP by immunofluorescence staining. Astrocytes colabeled with C3 and GFAP showed slight hypertrophy of the cell body and an increased number and length of cytoplasmic protrusions; conversely, astrocytes labeled with S100A10 showed few cytoplasmic protrusions (Figures 4D, E). Interestingly, the morphology of C3-labeled astrocytes was more similar to that of reactive astrocytes labeled in db/db mice, as shown in Figure 3E. This illustrates, at least in part, the conversion of a substantial number of reactive astrocytes to the A1 phenotype in the hippocampus of db/db mice.

In this experiment, LIRA was confirmed to effectively regulate the protein expression of C3 and S100A10 in the hippocampus of db/db mice, and the observed improvement of the astrocyte morphology. Thus, we preliminarily confirmed that LIRA treatment reduced the hyperactivation of astrocytes and the transformation of A1 astrocytes in the hippocampus of T2DM mice and maintained a certain level of A2 astrocytes.

### 3.5. LIRA attenuates extensive inflammatory infiltrates, including the hippocampus, in db/db mice

**FIGURE 5:**
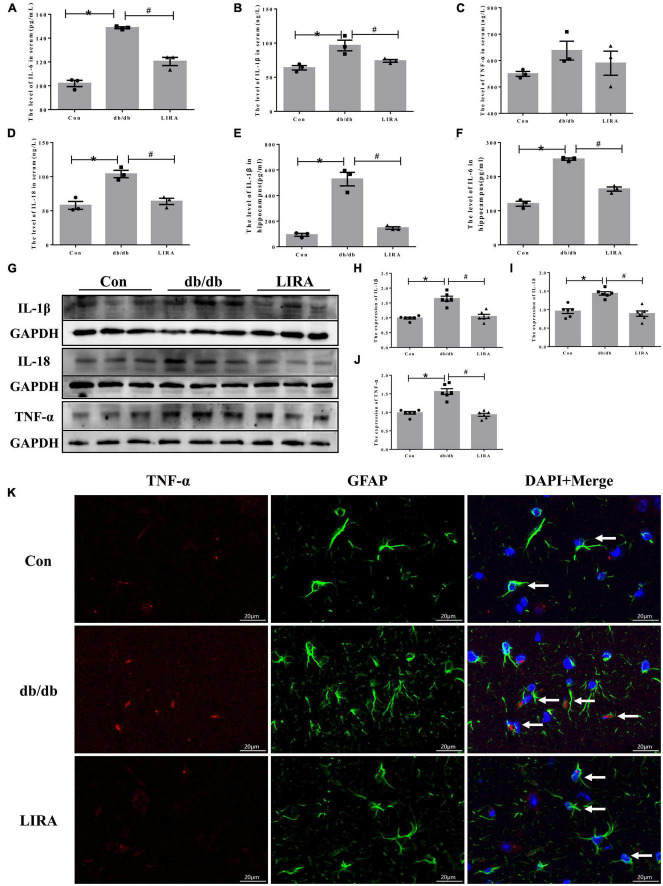
Liraglutide attenuates hippocampal neuroinflammation in db/db mice. Serum IL-6 **(A)**, IL-1β **(B)**, TNF-α **(C)**, and IL-18 **(D)** contents (*n* = 3). Hippocampal IL-1β **(E)** and IL-6 **(F)** contents (*n* = 3). **(G–J)** The expression and statistics of IL-1β, IL-18, and TNF-α proteins in the hippocampus (*n* = 6). **(K)** Immunofluorescence double-labeled staining of TNF-α/GFAP in the hippocampus, as indicated by the arrows (scale bar: 20 μm, *n* = 3). The data are shown as the means ± SEMs. *p < 0.05 vs. Con group, ^#^*p* < 0.05 vs. the db/db group.

Next, we assessed the level of neuroinflammation in T2DM. Using ELISA kits, we detected the expression of IL-6, IL-1β, TNF-α, and IL-18 in the serum of each group of mice, as well as the expression of IL-6 and IL-1β in the hippocampus, which are common pro-inflammatory factors. The expression levels of IL-6, IL-1β, and IL-18 were significantly increased in db/db mice, and this was reversed by LIRA (Figures 5A–F).

Furthermore, we evaluated the expression of IL-1β, IL-18, and TNF-α in the hippocampus of db/db mice by Western blotting. The expression levels of IL-1β, IL-18, and TNF-α in the hippocampus of db/db mice were significantly increased (Figures 5G–J). LIRA reduced the secretion of the above inflammatory factors (Figures 5G–J). Notably, by immunofluorescence colabelling of TNF-α with GFAP, we observed TNF-α expression on a proportion of astrocytes in the hippocampus of db/db mice, and this proportion was reduced after LIRA intervention (Figure 5K). In short, LIRA effectively inhibited hippocampal inflammation in db/db mice.

### 3.6. LIRA reduced iron deposition in the hippocampus and iron transport in astrocytes of db/db mice

**FIGURE 6:**
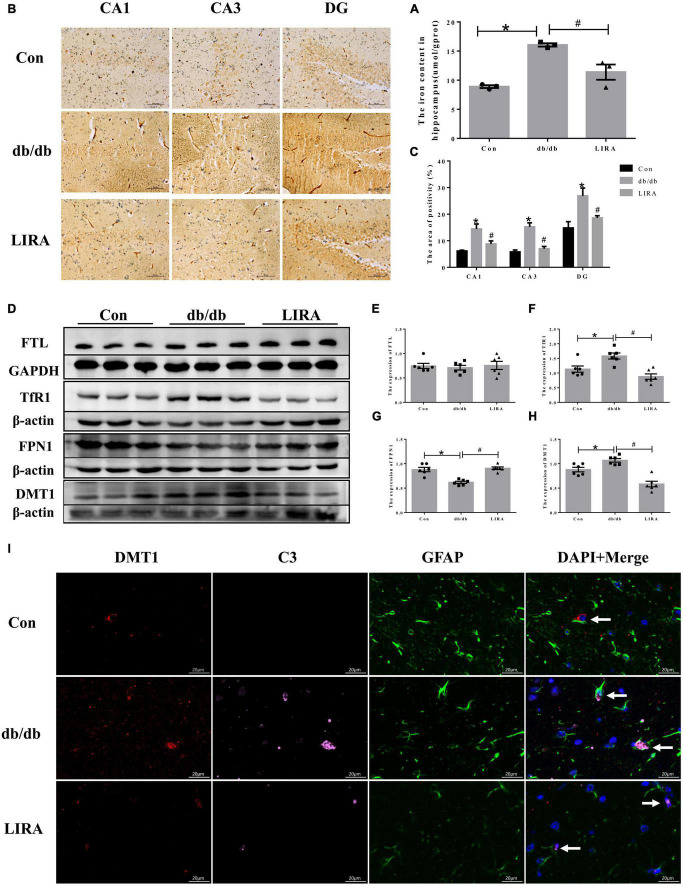
Liraglutide reduced iron deposition in the hippocampus and iron transport of db/db mice. **(A)** Hippocampal iron content (*n* = 3). **(B)** Perls’ staining in different parts of the hippocampus (*n* = 3). **(C)** The iron-positive area in the whole hippocampus. **(D–H)** The expression and statistics of FTL, TfR1, FPN1, and DMT1 proteins in the hippocampus (*n* = 6). **(I)** Immunofluorescence triple-labeled staining of DMT1/C3/GFAP in the hippocampus, as indicated by the arrows (scale bar: 20 μm, *n* = 3). The data are shown as the means ± SEMs. *p < 0.05 vs. Con group, ^#^*p* < 0.05 vs. the db/db group.

Aging and neurodegenerative diseases are associated with brain iron deposition (Rouault, 2013), and increased iron levels lead to reduced neuronal protection by peripheral astrocytes (Ci et al., 2020). Therefore, we used a tissue iron assay kit and Perls’ staining to determine the content and distribution of iron in the hippocampus. The iron levels were significantly increased in the hippocampus of db/db mice, encompassing CA1, CA3, and DG regions (Figures 6A–C). Furthermore, in the db/db group, the iron uptake protein TfR1 and the ferrous uptake protein DMT1 were increased; only the iron releasing protein FPN1 was downregulated, and the expression of the iron storage protein FTL in the hippocampus of the db/db mice did not change (Figures 6D–H). Our results showed that LIRA treatment attenuated the increase in hippocampal iron content, decreased TfR1 and DMT1 protein levels and increased FPN1 protein levels (Figures 6A–H). These results suggest that T2DM can lead to iron overload, which can be ameliorated by LIRA.

Notably, we also examined whether hippocampal iron deposition in db/db mice maintains enhanced A1 polarization in astrocytes. The brain tissue sections of each group were treated with triple immunofluorescence of DMT1, C3, and GFAP. The overall density of DMT1/C3/GFAP overlapping structures in the hippocampal CA1 region seemed to be higher in db/db mice, which may suggest that enhanced iron transport and accumulation are related to the polarization of A1 astrocytes (Figure 6I). Interestingly, LIRA treatment significantly reduced DMT1 expression in the hippocampus of db/db mice, accompanied by a decrease in C3 and GFAP expression (Figure 6I).

### 3.7. LIRA reduces the production of ROS and lipid peroxides in the hippocampus of db/db mice

**FIGURE 7:**
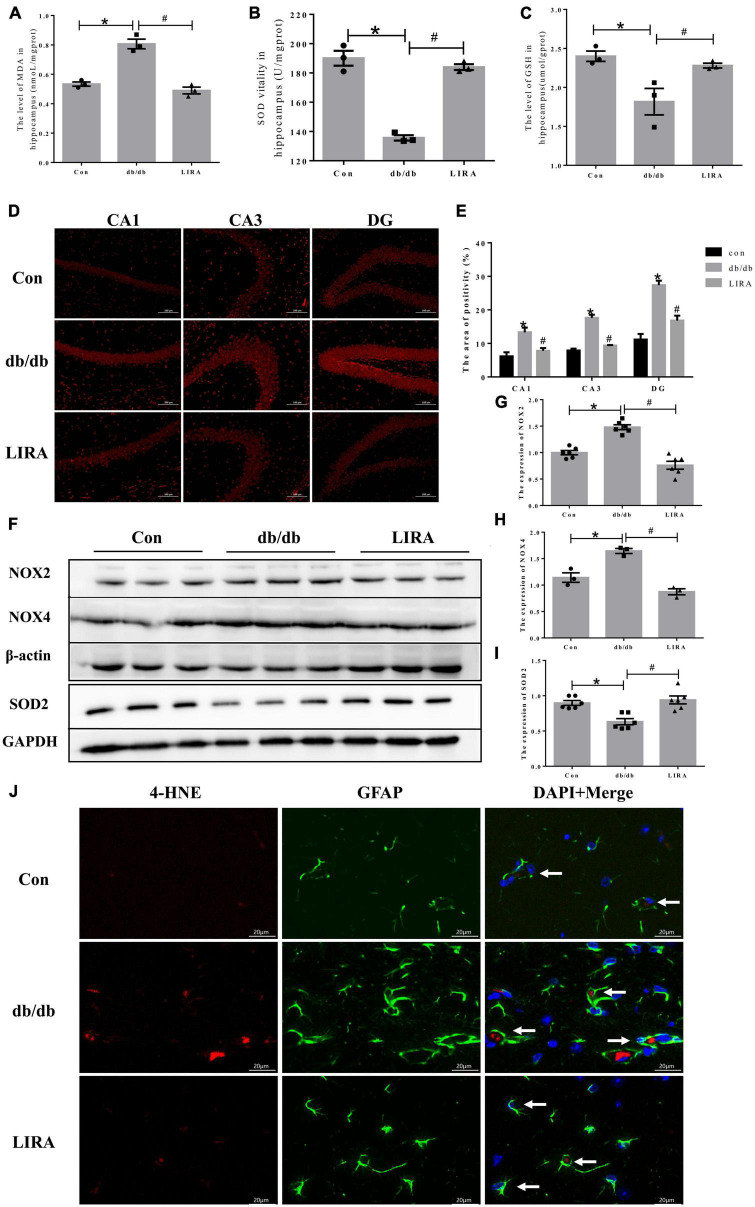
Liraglutide reduces the production of ROS and lipid peroxides in the hippocampus of db/db mice. Hippocampal MDA **(A)**, SOD **(B)**, and GSH **(C)** contents (*n* = 3). **(D)** Representative images of DHE staining in different regions of the hippocampus (scale bar: 100 μm, *n* = 3). **(E)** The positive area, as shown in panel **D)**. **(F–I)** The expression and statistics of NOX2, SOD2 (*n* = 6), and NOX4 (*n* = 3) proteins in the hippocampus. **(J)** Immunofluorescence double-labeled staining of 4-HNE/GFAP in the hippocampus, as indicated by the arrows (scale bar: 20 μm, *n* = 3). The data are shown as the means ± SEMs. *p < 0.05 vs. Con group, ^#^*p* < 0.05 vs. the db/db group.

Iron overload leads to the generation of ROS and lipid peroxides and triggers memory deficits. We assessed the beneficial effects of LIRA against oxidative stress. ELISA results showed that MDA was increased in the hippocampus of db/db mice, while the antioxidants SOD and GSH were decreased significantly (Figures 7A–C). DHE staining showed that ROS levels were significantly increased in the hippocampus of db/db mice (Figures 7D, E). LIRA treatment reduced the level of ROS and lipid peroxides induced by T2DM (Figures 7A–E).

To further elucidate the underlying mechanism of ROS generation, we examined NADPH oxidase, a key enzyme of redox signaling *in vivo* and a major source of ROS (Bruce-Keller et al., 2010). NOX2 and NOX4 protein levels were significantly increased in the hippocampus of db/db mice. Compared with the db/db group, the expression levels of NOX2 and NOX4 in the brains of LIRA-treated mice were significantly reduced, while the low expression of SOD2, an important enzyme against oxygen free radical-mediated tissue damage, was restored in LIRA-treated mice (Figures 7F–I). 4-HNE is an important marker of lipid peroxidation, and increased levels of 4-HNE in brain tissue eventually lead to neurodegenerative diseases (Li et al., 2022). The results showed that the expression level of 4-HNE was significantly upregulated in the hippocampus of db/db mice and partially overlapped with the expression of GFAP (Figure 7J). Treatment with LIRA reduced the high expression of 4HNE. This finding suggests a degree of homology between ROS and lipid peroxide production and astrocyte pathology, which are effectively protected by LIRA.

## 4. Discussion

The pathogenesis of cognitive impairment in DM is not fully understood, and elucidation of the underlying glial abnormalities may improve therapeutic strategies. This study assessed changes in neuroinflammation in db/db mice, particularly hyperpolarization of A1/A2 reactive astrocytes. These changes may be related to oxidative damage caused by iron overload in astrocytes. This study may further reveal the intervention of liraglutide on cognitive impairment in DM, which is specifically reflected in the improvement of the abovementioned changes in astrocytes.

Glycolipid metabolism disorder is a common symptom of DM complications. Glycogen metabolism is affected by changes in blood glucose concentration, and DM-induced excess glycogen deposition has been demonstrated in multiple organs, including the hippocampus (Shearer et al., 2011; Shima et al., 2017). DM causes metabolic effects to astrocytes, and one of the manifestations is increased storage of glucose in the form of glycogen (Cataldo and Broadwell, 1986; Garcia-Espinosa et al., 2003). Our study showed mildly elevated glycogen levels in the hippocampus of db/db mice, which may be a consequence of hyperglycemia, although the difference was not significant. LIRA decreased the content of glycogen in the hippocampus of db/db mice as a consequence of its effective regulation of glycolipid levels.

In previous studies, we paid more attention to the effect of LIRA on the synapses of hippocampal neurons in diabetic models (PSD95, synaptophysin, etc.) (An et al., 2022). In this study, we further confirmed neuronal damage in db/db mice, as evidenced by the expression of NeuN. NeuN expression was increased by LIRA, once again showing its neuroprotective effect. In fact, there is no doubt about the role of LIRA in regulating blood glucose, but it has been reported that the neuroprotective effect of LIRA does not mainly lie in the normalization of blood glucose in T2DM rats (Filchenko et al., 2018).

As mentioned earlier, reactive astrocytes proliferate in the T2DM brain, which is associated with cognitive impairment and inflammatory activation. Previous studies demonstrated that LIRA reduced palmitate-induced astrogliosis in the DG and CA1 region of the hippocampus (Barreto-Vianna et al., 2017). In this study, we found that LIRA attenuated the high expression of GFAP and the abnormal morphology of astrocytes in the hippocampus of db/db mice; thus, in the proportion of reactive astrocytes was decreased. Interestingly, whether astrocytes proliferate or decrease in the brains of diabetic models is still controversial (Coleman et al., 2004; Revsin et al., 2005; Nagayach et al., 2014; Faheem and El Askary, 2017; Li et al., 2018; Ribeiro et al., 2021). In all of those studies, the analysis of the number and morphology of astrocytes was reflected in the expression of GFAP. Due to the great differences in the morphology of astrocytes, this quantification is not accurate, and more accurate quantification methods are needed.

This study focused on the regulatory effect of LIRA on the phenotypic transformation of “A1” and “A2” reactive astrocytes in the hippocampus of a T2DM model. The increase in A2 astrocytes upregulated the secretion of neurotrophic factors, including BDNF, that support neuronal development, plasticity, and survival (Zha et al., 2022). Our study showed that LIRA increased the expression of BDNF in the hippocampus of db/db mice. Considering that LIRA may increase the number of A2-responsive astrocytes, characterized by an increase in S100A10, we believe that this may partly explain the increase in BDNF.

Conversely, the number of A1 astrocytes in the brain is often increased in neurodegenerative diseases (Liddelow et al., 2017). A1-reactive astrocytes are induced by IL-1β, TNF-α, and IL-18, and they can partially kill or inhibit neuronal activity by releasing inflammatory factors, such as TNF-α(Kwon and Koh, 2020; Xu et al., 2020; Fan and Huo, 2021). We suggest that activation of type A1 astrocytes in the hippocampus of db/db mice may exacerbate neuroinflammation, given the strong correlation between the two. In fact, studies have confirmed that LIRA has a good inhibitory effect on the secretion of inflammatory factors in the hippocampus (Cui et al., 2022). Our study confirmed that LIRA reduced A1-reactive astrocytes and neuroinflammation by downregulating the expression of C3, IL-1β, IL-18, and TNF-α. However, the function of astrocytes has not been fully revealed, so a simple dichotomous classification cannot reflect the various phenotypes of astrocytes, as mentioned by Kwon and Koh (2020).

**FIGURE 8:**
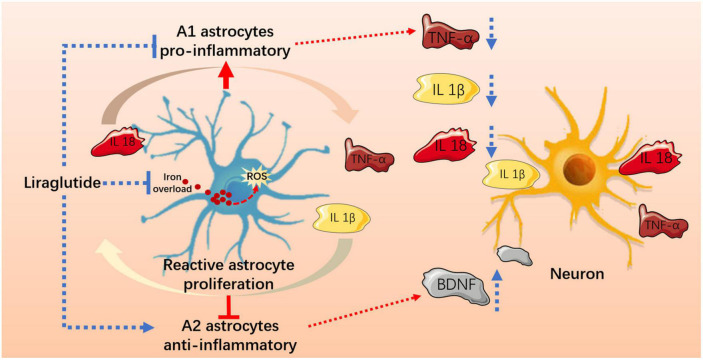
Liraglutide effectively improved iron metabolism and oxidative damage, regulated reactive astrocyte proliferation and A1/A2 typing, and reduced hippocampal neuroinflammation and neuronal damage in T2DM mice.

Cognitive impairment and brain damage in DM are linked to inflammation and imbalances in iron homeostasis. Cellular iron homeostasis is impaired during neuroinflammation, which leads to iron accumulation; in turn, excessive iron changes the inflammatory phenotype and causes secretion of diffusible inflammatory factors in microglia and infiltrating macrophages (Urrutia et al., 2021). Iron imbalance may induce changes in the maturation stage of astrocytes (Cheli et al., 2020). There is evidence of increased iron content in astrocytes in brain tissue from aging models (Connor et al., 1990). It has also been reported that upregulated DMT1 expression in an inflammatory environment leads to iron accumulation, further promoting astrocyte activation (Pelizzoni et al., 2013). These results confirm the effect of iron overload on astrocyte status. In fact, the main pathological consequence of iron overload is oxidative stress. Iron loading promoted O_2_(−) production and increased the expression of NADPH oxidase and the production of lipid peroxides, accompanied by excessive consumption of antioxidants (Marques et al., 2015; An et al., 2022). Interestingly, mitochondrial-derived oxidative stress resulted in A1-specific astrocyte polarization (Cao et al., 2021). We observed significant upregulation of TfR1 and DMT1 and downregulation of FPN1, which confirmed increased iron uptake and iron overload. Furthermore, we noticed that the activation of A1-responsive astrocytes may be associated with iron overload and oxidative stress, as deduced by the high overlap of DMT1/C3 elevation and multiple oxidative imbalances of peroxides. Fortunately, LIRA can effectively address excess iron and oxidative imbalance in the hippocampus, which may indirectly improve the polarization of A1 astrocytes (Figure 8).

This study has some limitations. First, our research results are only derived from animal model experiments, and the conclusion of this preliminary study needs to be further confirmed in cell experiments to afford it greater credibility. Second, we simply focused on the pathological changes in astrocytes. Indeed, there was a marked upregulation of proinflammatory pathways and activation of microglia in the brain tissue of DM mice, resulting in marked memory deficits (Rom et al., 2019). Given the cascading effects of glial cells, it is interesting to investigate the role of crosstalk between microglia and astrocytes in diabetic CNS inflammation (Sacristan, 2020). Third, we did not focus on the specific role of astrocyte polarization. As mentioned above, astrocytes are an important component of the blood-brain barrier, and the polarization of astrocytes in the T2DM brain may lead to the breakdown of the blood-brain barrier, but additional research is needed to confirm this. Finally, this study focused on the abnormal transport of iron in astrocytes in the hippocampus but lacked in-depth research on the cause of abnormal iron transport. Some reports emphasize that the increase in inflammatory-stimulated iron modulators and the activation of IRP1 can lead to cell iron overload, leading to neuronal death (Urrutia et al., 2021). Further exploration of the changes in iron metabolism in astrocytes in subsequent studies is warranted.

## 5. Conclusion

Here, we provide evidence that neuroinflammation and abnormal astrocyte A1/A2 polarization occur in the hippocampus of db/db mice, which may be related to iron overload-induced oxidative stress, also leads to neuronal damage. Liraglutide can effectively improve iron metabolism and oxidative damage, regulate the levels of A1/A2 reactive astrocytes and neuroinflammation, and alleviate cognitive impairment in T2DM.

## Data availability statement

The original contributions presented in this study are included in the article/supplementary material, further inquiries can be directed to the corresponding authors.

## Ethics statement

This animal study was reviewed and approved by the Medical Ethics Animal Care and Use Committee of Liaoning University of Traditional Chinese Medicine.

## Author contributions

YS, Y-FY, J-RA, J-TL, and Q-FW: conceptualization. J-RA and X-MG: methodology. J-RA, X-MG, and J-TL: resources. J-RA, X-MG, G-YS, J-NS, CZ, and J-XY: data curation. J-RA: writing—original draft. YS and Y-FY: writing—review and editing. J-RA, Q-FW, and X-MG: visualization. YS, Y-FY, and Q-FW: funding acquisition. All authors contributed to the article and approved the submitted version.
